# Occupational exposures and non-Hodgkin's lymphoma: Canadian case-control study

**DOI:** 10.1186/1476-069X-7-44

**Published:** 2008-08-07

**Authors:** Chandima P Karunanayake, Helen H McDuffie, James A Dosman, John J Spinelli, Punam Pahwa

**Affiliations:** 1 Canadian Centre for Health and Safety in Agriculture, University of Saskatchewan, Saskatoon, SK, Canada; 2 Cancer Control Research, British Columbia Cancer Agency, British Columbia, Canada; 3 Department of Community Health & Epidemiology, University of Saskatchewan, Saskatoon, SK, Canada

## Abstract

**Background:**

The objective was to study the association between Non-Hodgkin's Lymphoma (NHL) and occupational exposures related to long held occupations among males in six provinces of Canada.

**Methods:**

A population based case-control study was conducted from 1991 to 1994. Males with newly diagnosed NHL (ICD-10) were stratified by province of residence and age group. A total of 513 incident cases and 1506 population based controls were included in the analysis. Conditional logistic regression was conducted to fit statistical models.

**Results:**

Based on conditional logistic regression modeling, the following factors independently increased the risk of NHL: farmer and machinist as long held occupations; constant exposure to diesel exhaust fumes; constant exposure to ionizing radiation (radium); and personal history of another cancer. Men who had worked for 20 years or more as farmer and machinist were the most likely to develop NHL.

**Conclusion:**

An increased risk of developing NHL is associated with the following: long held occupations of faer and machinist; exposure to diesel fumes; and exposure to ionizing radiation (radium). The risk of NHL increased with the duration of employment as a farmer or machinist.

## Background

Non-Hodgkin's Lymphoma (NHL) is a cancer of the lymphatic system [1,2]. Even though NHL is a relatively rare disease, its incidence rates have been increasing worldwide for both men and women. The incidence rates in Canada, for both males and females were increased by about 50% between 1978 and the late 1990s. After the latter time, incidence rates have stabilized. Mortality rates of NHL have followed a similar pattern [3]. Age-standardized rates have increased faster among males than among females [1-5]. A number of factors, including inherited and acquired immunodeficiency states [6] as well as infectious, physical, and chemical agents have been associated with an increased risk for NHL [6,7].

Epidemiological studies have reported positive associations between NHL and certain occupations including those of farmers [8-16], pesticide applicators [12,17-20], drivers [21,22], and managers [23,24]. Several studies have reported no association between development of NHL and the agricultural occupations (farmers, agricultural and forestry workers and pesticide applicators [25-27]). Occupational exposures of a priori interest include pesticides [28-33], dusts (metal, wood, paper [8], etc), paints [8,35], diesel exhaust fumes [21,22,34,35], cleaning fluids [8], cutting oils [36], and solvents [37,38]. In this paper, we examined the association between NHL and (1) selected long term occupations, and (2) occupational exposures based on an individual's occupational history, and (3) duration of employment.

## Methods

Details of the study design and methodology have been previously published [39-41]. Briefly, we conducted a six province Canadian population based case-control study of men with an incident first diagnosis of NHL between 1991 to 1994; control subjects were frequency matched by age ± 2 years to be comparable with the age distribution of the entire case group (Soft Tissue Sarcoma (STS), Hodgkin's Disease (HD), NHL, and Multiple Myeloma (MM)) within each province of residence. The study had approximately three matched controls for each NHL case. Deceased subjects were ineligible as either cases or controls. All participating control subjects were used in the statistical analysis of each cancer site. Cases were identified from provincial cancer registries – except in Quebec where hospital records were used – and were coded using ICD-O 2^nd ^edition except Quebec which used ICD-O 1^st ^edition [42]. Malignant morphology codes 9591, 9642, 9670–9764, and 9823 were included. A reference pathologist reviewed the tumour tissue slides for 60% of the NHL cases, and confirmed NHL in all but 2% of cases. Cases not confirmed as NHL were eliminated. Control subjects were identified through provincial health insurance programs except in Ontario (telephone listing) and British Columbia (voter's lists), as generally described [39-41].

The study design consisted of two stages: Stage 1 was a self-administered postal questionnaire; and Stage 2 was a detailed pesticide exposure information collected via telephone interview. With permission, we modified a pesticide exposure questionnaire developed by Hoar et al. [43] to create the study questionnaire. The results in this manuscript are based on the Stage 1 postal questionnaire only.

The postal questionnaire captured demographic details, personal medical history, lifetime occupational history and specific occupational exposures of interest. Occupational information included a list of all full time jobs held by the respondent for at least one year. For each job held, we collected information on job titles, business organization – whether service or industry – and duration of employment. A list of occupational exposures that have been epidemiologically linked to NHL or to one of the other three types of cancers which we studied simultaneously was grouped into dusts, coal products, printing products, paints, metals, pesticides, radiation and miscellaneous. Additional details of exposure to agricultural chemicals in broad classes i.e. herbicides, fertilizers etc, were obtained. Job titles and each industry's coding were provided by Statistics Canada [44].

### Statistical analysis

Data were entered into a custom designed SPSS-data entry program. Results were presented as frequencies for categorical variables; mean, standard deviation (SD) for continuous variables for cases and controls were presented separately. We obtained information about the duration of employment (measured in years) for each individual. The occupations were selected for analysis if the occupant worked in a particular occupation at least for one year and at least 2% of cases for that occupational category. Based on that information, we derived two new variables called ever held occupations and long held occupations. Occupations were defined as ever held occupation if respondents worked at least for one year in that occupation. Occupations were defined as long held occupation if respondents worked for 10 years or more in that occupation. Duration of employment is the total of number of years in each long held occupation. A bivariate analysis was conducted to determine the association between each explanatory variable and the NHL outcome. Based on this model, building procedure explanatory variables with p < 0.20 were selected for the multivariate model. Statistically significant (p = 0.05) variables and important explanatory variables were considered for the final multivariate model adjusting for age and province of residence. Conditional logistic regression was used to compute adjusted odds ratios (OR) and 95% confidence intervals (95% CI).

### Ethics

The letters of informed consent, questionnaires, and all other correspondence with study participants were approved by the relevant ethics agencies in each province. All of the information that could be used to identify study participants remained within each province of origin under the supervision of the provincial principal investigators.

## Results

This study includes responses from 513 cases with NHL and 1506 control subjects. The mean age ± standard deviation (SD) of cases was 57.7 ± 14.0 years and, of the controls, 54.1 ± 16.0 years. More cases (n = 74, 14.4%) than controls (n = 87, 5.8%) had a personal history of cancer other than NHL (OR_adj _(95 % CI): 2.56 (1.81, 3.62)). There were no significant differences between NHL cases and controls with respect to their education level and to whether they ever lived or worked on a farm. Results are shown in Table 1.

**Table 1:** Characterization of study participants stratified by NHL case- control status: demographics and selected medical history

	NHL (*N = 513*)	Controls (*N = 1506*)	OR^b^_adj _[P1](95% CI)
**Demographics**			
Mean age ± SD (years)	57.7 ± 14.0	54.1 ± 16.0	
Education Level^a^			
University and Vocational	28 (6.6)	96 (5.5)	1.23 (0.81, 1.88)
University	94 (18.5)	310 (20.8)	1.08 (0.68, 1.70)
Vocational	111 (21.9)	358 (24.1)	1.06 (0.67, 1.70)
Elementary/High school	274 (54.0)	723 (48.6)	1.00
Ever lived/worked on a farm			
Yes n (%)	235 (45.8)	673 (44.7)	1.02 (0.82, 1.27)
No n (%)	278 (54.2)	833 (55.3)	1.00

**Medical History**			
Previous diagnosis of Cancer			
Yes n (%)	74 (14.4)	87 (5.8)	**2.56 (1.81, 3.62)**^c^
No n (%)	439 (85.6)	1419 (94.2)	1.00

^a ^25 missing

^b ^Adjusted for age (5 year groups) and province

^c ^Statistically significant results are bold.

Table 2 shows the distribution of ever held occupations and long held occupations during a lifetime stratified by case-control status. None of the ever held occupations were statistically significant. The long held occupations (10 years or more) as farmer and machinist showed a significant risk increase for NHL. The adjusted odds ratios (OR_adj_) and 95% confidence intervals (95% CI) for a long held occupation during the lifetime as farmer and machinist were 1.54 (1.05, 2.27) and 2.21 (1.02, 4.79) respectively. Using four categories (no exposure, < 10 years, 10–20 years, and > 20 years), further models with years in these industries were used to investigate whether or not there is a dose-response relationship between the long held occupation as a farmer and a machinist and NHL (Table 3). A dose-response relationship between duration of exposure as farmer and incidence of NHL was observed. Those who worked as a farmer for more than 20 years were 1.5 times more likely to be diagnosed with NHL than non-exposed subjects. Similarly, we observed a dose-response relationship between duration of exposure as a machinist and incidence of NHL. Those who worked as a machinist for more than 20 years were 2.3 times more likely to be diagnosed with NHL than non-exposed subjects (Table 3).

**Table 2:** Adjusted odds ratio (OR) and 95% confidence interval (95% CI) for different occupations (job titles).

Job Title (code#)	NHL cases n (%)	Controls n (%)	OR _adj_ a (95% CI)
**Ever held Occupations**			
Accountant (1)	30 (5.8)	81 (5.4)	1.21 (0.77, 1.89)
Administrator (2)	11 (2.1)	52 (3.4)	0.58 (0.30, 1.15)
Carpenter (12)	21 (4.1)	55 (3.6)	1.06 (0.63, 1.79)
Clerk (17)	14 (2.7)	92 (6.1)	0.44 (0.24, 0.79)
Constructor (19)	14 (2.7)	78 (5.2)	0.51 (0.28, 0.93)
Driver (25)	55 (10.7)	133 (8.8)	1.29 (0.91, 1.82)
Electrician (26)	16 (3.1)	47 (3.1)	0.99 (0.54, 1.78)
Engineer (27)	13 (2.5)	68 (4.5)	0.54 (0.29, 1.02)
Factory worker (29)	13 (2.5)	46 (3.0)	1.14 (0.59, 2.17)
Foreman (30)	11 (2.1)	39 (2.6)	0.64 (0.32, 1.28)
Farmer (31, 33, 89)	86 (16.7)	230 (15.3)	1.14 (0.85, 1.54)
Armed forces (138)	28 (5.5)	92 (6.1)	0.76 (0.48, 1.18)
Janitor (41)	14 (2.7)	40 (2.7)	1.07 (0.57, 2.02)
Labourer (44)	31 (6.0)	99 (6.6)	0.86 (0.56, 1.33)
Lumberman (46)	17 (3.3)	38 (2.5)	1.12 (0.61, 2.03)
Machinist (47)	22 (4.3)	49 (3.2)	1.41 (0.83, 2.40)
Manager (48)	63 (12.3)	183 (12.1)	0.97 (0.70, 1.33)
Mechanic (49)	26 (5.1)	88 (5.8)	0.83 (0.52, 1.31)
Salesman (73)	44 (8.6)	127 (8.4)	1.06 (0.73, 1.53)
School Teacher (74)	31 (6.0)	88 (5.8)	0.96 (0.62, 1.48)
Welder (86)	13 (2.5)	33 (2.2)	1.25 (0.64, 2.44)
Office worker (97)	17 (3.3)	68 (4.5)	0.70 (0.40, 1.22)
Equipment hander (134)	14 (2.7)	37 (2.5)	1.34 (0.70, 2.56)

**Long held Occupations**			
Accountant (1)	20 (3.9)	41 (2.7)	1.39 (0.79, 2.42)
Driver (25)	27 (5.3)	48 (3.2)	1.45 (0.88, 2.37)
Farmer (31, 33, 89)	50 (9.8)	106 (7.0)	**1.54 (1.05, 2.27)**^c^
Machinist (47)	12 (2.3)	16 (1.1)	**2.21 (1.02, 4.79)**^c^
Manager (48)	31 (6.0)	96 (6.4)	0.86 (0.56, 1.32)
Mechanic (49)	15 (2.9)	49 (2.2)	1.00 (0.99, 1.02)

^# ^Statistics Canada. Standard occupational classification. Ottawa: Minister of Supply and Services, 1980.

^a^ All odds ratios were adjusted for age and province of residence.

^c ^Statistically significant results are bold.

**Table 3:** Duration of exposure as a farmer and machinist and risk of NHL

Duration (in years)	NHL (*N = 513*)	Control (*N = 1506*)	OR (95% CI)^a^
	n (%)	n (%)	
**Job Title: Farmer**			
No exposure	427 (83.2)	1276 (84.7)	1.00
<10 years	36 (7.0)	124 (8.2)	0.84 (0.51, 1.41)
10–20 years	7 (1.4)	23 (1.5)	1.40 (0.57, 3.43)
> 20 years	43 (8.4)	83 (5.5)	**1.55 (1.02, 2.36)**^c^

**Job Title: Machinist**			
No exposure	491 (95.7)	1457 (96.7)	1.00
<10 years	10 (1.9)	33 (2.2)	0.75 (0.30, 1.88)
10–20 years	2 (0.4)	4 (0.3)	1.77 (0.31, 10.22)
> 20 years	10 (1.9)	12 (0.8)	**2.33 (1.00, 5.52)**^c^

^a ^all odds ratios were adjusted for age and province of residence.

^c ^Statistically significant results are bold.

Of the 45 specific occupational exposures grouped into six classes (dusts, coal products, printing, paints, metals and miscellaneous), only exposure to diesel exhaust fumes showed an association with NHL (Table 4). Ever exposure to solvents and exposure to wood or paper dust were not associated with NHL. Ever exposure to ionizing radiation (radium) showed a significant association with the risk of NHL incidence (OR adj (95% CI): 3.26 (1.38, 7.73)).

**Table 4:** Adjusted odds ratio (OR) and 95% confidence interval (95% CI) for different occupational exposures.

	NHL (*N = 513*)		Control (*N = 1506*)		
			
Exposure	n^b^	%	n^b^	%	OR_adj _(95% CI)^a^
**Dusts**					
Cement dust	134	26.1	432	28.7	0.93 (0.73, 1.18)
Fiberglass dust	102	19.9	319	21.2	1.02 (0.78, 1.33)
Coal dust	63	12.3	149	9.9	1.19 (0.86, 1.66)
Soil/field dust	142	27.7	375	24.9	1.26 (0.99, 1.61)
Whey dust	12	2.3	38	2.5	0.89 (0.45, 1.77)
Paper dust	68	13.3	180	11.9	1.22 (0.89, 1.67)
Wood dust	143	27.9	445	29.5	0.95 (0.75, 1.20)
Coke dust	10	1.9	58	3.8	0.53 (0.26, 1.06)
Stone dust	55	10.7	173	11.5	0.99 (0.71, 1.40)
Grain Dust	117	22.8	347	23.0	0.99 (0.76, 1.29)
Sand	90	17.5	303	20.1	0.89 (0.67, 1.16)
Cardboard dust	50	9.7	170	11.3	1.01 (0.71, 1.44)
Metal dust	120	23.4	368	24.4	1.06 (0.82, 1.36)

**Coal Products**					
Pitch	17	3.3	38	2.5	1.24 (0.68, 2.25)
Asphalt	46	8.9	142	9.4	0.96 (0.67, 1.38)
Crude petroleum	30	5.8	84	5.6	1.00 (0.64, 1.57)
Tar/tar products	53	10.3	143	9.5	1.20 (0.84, 1.69

**Printing**					
Printing inks	35	6.8	134	8.9	0.90 (0.60,1.36)
Printing fluid	28	5.5	96	6.4	0.93 (0.59, 1.47)

**Paints**					
Paints, dyes	148	28.8	442	29.3	1.06 (0.84, 1.33)

**Metals**					
Arsenic	13	2.5	28	1.9	1.45 (0.72, 2.93)
Nickel	29	5.6	85	5.6	1.11 (0.71, 1.74)
Cadmium	20	3.9	55	3.6	1.07 (0.62, 1.84)
Zinc	38	7.4	103	6.8	1.12 (0.75,1.67)
Mercury	20	3.9	63	4.2	0.84 (0.49, 1.43)
Chromium	24	4.7	58	3.8	1.33 (0.79, 2.22)
Iron	40	7.8	100	6.6	1.18 (0.79, 1.77)
Lead	65	12.7	182	12.1	1.03 (0.75, 1.42)
Aluminum	71	13.8	220	14.6	1.03 (0.76, 1.40)

**Miscellaneous**					
Asbestos	76	14.8	237	15.7	0.91 (0.68, 1.21)
Used motor oil	117	22.8	400	26.6	0.89 (0.69, 1.15)
Diesel exhaust fumes	183	35.7	464	30.8	**1.33 (1.06,1.67)**^c^
Cutting oils	74	14.4	277	18.4	0.81 (0.60, 1.08)
Cleaning fluids	124	24.2	419	27.8	0.93 (0.72, 1.19)
Preservatives	9	1.7	21	1.4	1.11 (0.49, 2.50)
Chlorine	68	13.3	202	13.4	1.05 (0.77, 1.43)
Hair permanent solutions	11	2.1	33	2.2	0.99 (0.48, 2.04)
Sour gas	24	4.7	92	6.1	0.69 (0.42, 1.12)
Wood smoke	121	23.6	371	24.6	0.95 (0.75, 1.22)
Lubricants	152	29.6	477	31.7	0.99 (0.78, 1.25)
Solvents	167	32.5	516	34.3	1.01 (0.80, 1.28)
Ether	51	9.9	170	11.3	0.88 (0.62, 1.25)
Mouldy grain/forage	61	11.9	176	11.7	1.09 (0.78, 1.53)
Hair dyes	15	2.9	33	2.2	1.33 (0.69, 2.52)
Cyanide	10	1.9	36	2.4	0.79 (0.38, 1.63)

**Non-ionizing radiation**					
Ultra Violet Light	44	8.6	151	10.0	1.06 (0.73, 1.55)
Horticultural Grow lights	12	2.3	39	2.59	0.91 (0.46, 1.79)
Unshielded microwaves	3	0.6	25	1.7	0.39 (0.11, 1.32)

**Ionizing radiation**					
Radium	12	2.34	12	0.80	**3.26 (1.38, 7.73)**^c^
Uranium	12	2.34	18	1.20	2.10 (0.97, 4.56)

^a ^all odds ratios were adjusted for age and province of residence.

^b ^n and % are given for the "yes" responses.

^c ^Statistically significant results are bold.

Table 5 shows the results of multivariate conditional logistic regression models for the long held jobs of farmer and machinist. The variables that remained statistically significantly associated with increased risk of NHL for long held job as a farmer were personal history of another cancer and exposure to ionizing radiation (radium). The variables for the long held job as a machinist associated with increased risk of NHL were personal history of another cancer, exposure to ionizing radiation (radium) and exposure to diesel. Duration of exposure for the long held jobs of farmer and machinist were borderline significant at 5% level (p = 0.08 and p = 0.059), but there was evidence of an increase risk of NHL with longer duration of exposure.

**Table 5:** Multivariate models of the important covariates associated with NHL for long held occupations.

Variable	Farmer	Machinist
	
	OR (95% CI)^a^	OR (95% CI)^a^
Personal history of another cancer (yes)	**2.60 (1.83, 3.69)**^c^	**2.57 (1.82, 3.65)**^c^
Ever exposed to ionizing radiation (radium) (yes)	**3.41 (1.44, 8.11)**^c^	**3.21 (1.34, 7.67)**^c^
Ever exposed to diesel (yes)	1.23 (0.97, 1.56)	**1.28 (1.02, 1.61)**^c^
		
Duration (reference to no exposure)		
<10 years	0.77 (0.45, 1.30)	0.73 (0.29, 1.86)
10–20 years	1.34 (0.54, 3.34)	1.87 (0.33, 10.57)
> 20 years	1.47 (0.95, 2.29)	2.34 (0.97, 5.68)

^a ^all odds ratios were adjusted for age and province of residence.

^c ^Statistically significant at 5% level results are bold.

## Discussion

Our study investigated the association between NHL and several occupations and occupational exposures. The findings revealed that two long held occupations (10 years or more), farmer and machinist, were significantly associated with increased risk of developing NHL. One of the possible explanations is that farmers and drivers might be exposed to pesticides and engine exhaust and machinists might be exposed to solvents or engine exhaust at the work place. The increased risk of NHL for farmer and machinist seen in our study is consistent with the findings from other studies [8-16].

Pesticides including herbicides and insecticides have been associated with Non-Hodgkin's Lymphoma in studies of farmers, agricultural related workers, other pesticide applicators, manufacturing workers and other exposed populations [39,45]. Grain handlers exposed to pesticides, grain dusts, and organic solvents were shown a five-fold risk of NHL [46]. Our study confirms that those who held the long held job title as a farmer (farmer, farm labourer and farm managers) had 1.5 times higher risk of being diagnosed with NHL than those who held a job title from the category of non-farmer.

Our results confirm previously reported associations of NHL and a personal history of cancer [47,48]. Occupational exposure to dust (wood, paper, metal etc.), coal products, paints, metal, and printing are unlikely to increase the risk of NHL, as is evident from our analysis. In contrast, Kawachi et al [49] found a significant association between working with wood and NHL. In addition, Kogevinas et al [50] found an increased risk of Lymphomas in pulp and paper workers. Ever exposure to diesel exhaust fumes is likely to increase the risk of NHL, as is evident from our analysis. Our finding is agreement for diesel exhaust fumes with Baris et al [21] and Maizlish et al [34].

The mechanism of cancer induction by radiation suggested in our study is not clear. The most widely accepted hypothesis is that some of the ionizing events, which occur when radiation is absorbed in tissue, produce a change in the genes or chromosomes of one or more cells [51]. A case-referent study conducted to investigate the possible association between occupation and occupational exposures and risk of hematological malignancies showed that exposure to asbestos, hydrocarbons, fertilizer, radiation, pesticides and mineral oils were highly associated with hematological malignancies [10]. Another matched case-control study in the nuclear industry [52] found no significant excess of NHL at any radiation exposure level. Archer [51] stated that uranium mill workers appeared to have excess Lymphomas. In our study, any form of radiation exposure at work was considered. Exposure to ionizing radiation (radium) is significantly associated with increase risk of NHL, which suggests equivocal evidence of an association with NHL presented by Ron [53].

There are many potential sources of non-ionizing radiation to workers. One of them is ultraviolet (UV) radiation. There is suggestive evidence that exposure to ultraviolet (UV) light, an established cause of immune suppression, may increase the risk of NHL [54-57]. The most recent epidemiologic literature suggests that there is no association or protective effect between exposure to sunlight and NHL [58-63]. Our study did not find any association between exposure to ultraviolet (UV) light with NHL.

Solvents have been associated with NHL in a number of studies [64-66], including studies of rubber workers [67], aircraft maintenance workers [68], and dry cleaners [69]. In particular, benzene exposure is common in above mention occupations and this may be due to its effects on the immune system [66]. Other occupations which might involve exposure to solvents or related chemicals and which are reported as being at increased risk of NHL include those of highway workers [34], petroleum refinery employees [70-72], styrene workers [73], chemists [74,75], and chemical manufacturers [76,77]. We could not find any association between NHL and exposure to solvents, cleaning fluids, or preservatives.

A major strength of this study is the large number of cases and controls from residents of six Canadian provinces. Questions were designed to obtain a complete occupational history and extensive list of potential occupational exposures. A reference pathologist validated 84% of the NHL tumours.

There are, however, several limitations in this study. One of the limitations is the potential for recall bias and misclassification of pesticide exposures. Also, occupational exposures in this study were self-reported and this might also bias results. Due to budget constraints, the study was restricted to males. The response rates of 67.1% for cases and 48% for controls represent another potential limitation that could create misleading conclusions if the non-respondents differ significantly from the respondents with respect to the variables under investigation. We compared non-respondents to respondents using postal codes as an indicator of rural residence and did not find a rural bias among respondents. The most common reasons for not participating were death, change of address, and refusal for both cases and controls. Another limitation was the possibility of false-positive findings given the large number of jobs and exposures assessed.

## Conclusion

Our results support previous findings of an association between NHL and specific job titles and occupational exposures. In our analysis, NHL was associated with personal history of cancer, exposure to diesel exhaust fumes, exposure to ionizing radiation (radium) and long held occupations such as farmer and machinist. Also, we have supportive evidence of increased risk of NHL with longer durations of exposure.

## Abbreviations

NHL: Non-Hodgkin's Lymphoma; ICD: International Classification of Diseases; STS: Soft Tissue Sarcoma; HD: Hodgkin's Disease; MM: Multiple Myeloma.

## Competing interests

The authors declare that they have no competing interests.

## Authors' contributions

CPK analyzed data and prepared the manuscript. HHM designed, coordinated the study and collect the data. JAD participated in study design, coordination, data collection and manuscript preparation. JJS participated in the design of the study and data collection. PP designed and coordinated the study as well as collected and prepared the manuscript.
